# The longitudinal mediating role of career compromise between psychological contract breach and turnover intention among newly recruited master of nursing graduates: a cross-lagged panel analysis

**DOI:** 10.3389/fpsyg.2026.1943987

**Published:** 2026-10-13

**Authors:** Rui Li, Qinhui Yu, Jing Peng, Yun Miao, Hong Song

**Affiliations:** 1 Chronic Disease Management Center, The Second Affiliated Hospital of Xuzhou Medical University, Xuzhou, Jiangsu, China; 2 Department of Nursing, The Second Affiliated Hospital of Xuzhou Medical University, Xuzhou, Jiangsu, China

**Keywords:** newly recruited, Master of Nursing, psychological contract breach, career compromise, turnover intention, longitudinal study, cross-lagged model, mediation effect

## Abstract

**Objective:**

To examine trajectories and temporal associations among psychological contract breach, career compromise and turnover intention in newly recruited Master of Nursing graduates, and to clarify the mediating role of career compromise.

**Methods:**

In this three-wave panel study, 295 Master of Nursing graduates newly recruited to 14 Chinese tertiary hospitals were assessed within the first month of employment (T1) and at 6 (T2) and 12 (T3) months. A cross-lagged panel model was fitted under maximum likelihood, with mediation tested by bootstrap (5,000 iterations).

**Results:**

All three variables increased significantly over 12 m (breach, 14.45 ± 2.26 to 15.90 ± 2.35; career compromise, 30.36 ± 5.74 to 34.75 ± 5.97; turnover intention, 15.24 ± 2.14 to 16.91 ± 2.36; all *P* < 0.001). The final model fitted well (χ^2^/df = 2.43, CFI = 0.985, TLI = 0.965, RMSEA = 0.022, SRMR = 0.036). Breach preceded later career compromise and turnover intention, and career compromise preceded later turnover intention (β = 0.16-0.26, all *P* < 0.01); all six reverse paths were small and non-significant (β = 0.04–0.08). Wald tests favored the hypothesized direction in all six reciprocal pairs, but the difference was significant in only one (breach → career compromise, T1 → T2: *P* = 0.049) and borderline in its T2 → T3 counterpart (*P* = 0.060). One indirect pathway was significant: PCB(T1) → CC(T2) → TI(T3) = 0.25 × 0.21 = 0.053 [95% CI (0.032, 0.112)]. A second pathway through compromise carried to T3 was not estimated: the within-wave association between compromise and intention is a residual covariance, not a directional path, and cannot enter an indirect effect. In the original metric this is about 0.08 point on the 6–24 intention scale, under 5% of the 1.67-point rise observed.

**Conclusion:**

Among newly recruited Master of Nursing graduates in Chinese tertiary hospitals, breach was longitudinally associated with higher turnover intention, partly through career compromise. Because the design is observational, these are temporal associations rather than causal effects, and the indirect effect is small. The findings identify a potentially modifiable early-career signal, not a basis for staffing policy; interventions on developmental promises merit controlled evaluation.

## Introduction

With the rapid advancement of higher nursing education in China, the Master of Nursing degree has emerged as a key qualification for advanced practice nurses equipped with both clinical competence and research capability. These individuals are expected to drive specialty nursing development, quality improvement, and disciplinary innovation (Zhuang et al., 2026; Zhu et al., 2025). However, multiple investigations have revealed that turnover rates among newly recruited Master of Nursing graduates are markedly higher than those of nurses with bachelor's or lower qualifications, with the peak departure period concentrated within the first year of employment (Li et al., 2021; Xu et al., 2025). This phenomenon represents a substantial drain on training resources and undermines the stability of nursing teams.

Psychological contract breach refers to an individual's cognitive appraisal that the organization has failed to fulfill implicit or explicit reciprocal obligations (Verma et al., 2025). Morrison and Robinson proposed that breach experiences act as critical antecedents to disappointment (Morrison and Robinson, 1997), distrust, and withdrawal behaviors. Master of Nursing graduates typically enter the workforce with clearly defined expectations regarding professional growth. When confronted with predominantly basic-care duties and ambiguous advancement pathways, they are highly susceptible to intense feelings of psychological contract breach, which in turn fuel intentions to leave (Yang et al., 2024).

Career compromise originates in Gottfredson's theory of circumscription and compromise (Gottfredson, 1981). The theory proposes that occupational preferences are progressively narrowed (circumscription) to a “zone of acceptable alternatives” compatible with the developing self-concept, and that when accessible options fall outside that zone individuals relinquish more preferred alternatives in favor of attainable ones (compromise). Compromise is therefore an accommodative, self-protective response to perceived constraint rather than a simple attitude, and it follows a characteristic order: interest is surrendered first, whereas prestige and sex-type are surrendered last (Gottfredson, 1981). Three conceptual boundaries matter here. Job crafting describes employees actively redrawing the task, relational and cognitive boundaries of their work (Wrzesniewski and Dutton, 2001); it is agentic and constructive, whereas career compromise is accommodative and entails downward revision of aspirations. Perceived over qualification is the judgement that one holds surplus education, experience or skills relative to job requirements (Maynard et al., 2006)—a person–job fit appraisal that may elicit compromise but is not itself the accommodation. Relative deprivation is the resentful appraisal that one is worse off than a relevant standard (Smith et al., 2012); it captures the affective-evaluative reaction to disadvantage, whereas career compromise captures the cognitive-behavioral adjustment made in response to it. In nursing research, career compromise has usually been operationalized with the multidimensional scale developed by Weng et al. (2018), which distinguishes developmental opportunity, career fit and social expectation compromise. Prior cross-sectional studies have suggested positive correlations among psychological contract breach, career compromise, and turnover intention (Yin et al., 2026; Lu et al., 2023), yet these investigations were limited by single-time-point designs that cannot rule out reverse or reciprocal temporal ordering, or capture the dynamic evolution of these associations over time.

Cross-lagged panel models offer a useful framework for separating autoregressive (stability) effects from cross-lagged effects, thereby clarifying the temporal ordering of associations among variables. Such models remain observational, however: even with three waves they cannot establish causality, and their estimates may conflate stable between-person differences with within-person change. Building on this methodology, the present study employed a three-wave longitudinal design with a sample of newly recruited Master of Nursing graduates. We constructed a cross-lagged panel model linking psychological contract breach, career compromise, and turnover intention to elucidate the temporal sequence through which turnover risk develops. The findings aim to inform evidence-based retention strategies for hospital administrators.

On the basis of the above, the following hypotheses were specified in advance and tested:

**H1:** Psychological contract breach, career compromise and turnover intention all increase across the first 12 m of employment.
**H2:** After autoregressive stability is controlled, psychological contract breach positively predicts subsequent career compromise (H2a) and subsequent turnover intention (H2b), and career compromise positively predicts subsequent turnover intention (H2c).
**H3:** Career compromise mediates the longitudinal association between psychological contract breach and turnover intention, such that higher breach at T1 is indirectly associated with higher turnover intention at T3 through higher career compromise at T2.
**H4:** (directional test, not a directional prediction): each hypothesized-direction cross-lagged coefficient is larger than its reverse-direction counterpart. This was evaluated as a formal test of equality for every reciprocal pair rather than assumed; the reverse paths were estimated without a predicted sign, and the outcome of all six tests is reported irrespective of whether it favors the hypothesized direction.

## Materials and methods

### Study design and participants

This longitudinal study adopted a three-wave panel design. Using convenience sampling, we recruited Master of Nursing graduates who had commenced employment between August and December 2024 at 14 tertiary-grade hospitals across China. Inclusion criteria were: (a) holder of a Master of Nursing degree with valid nursing licensure; (b) employed ≤ 1 month at the time of the baseline survey and entering clinical practice for the first time; (c) provision of informed consent and commitment to completing the full 12-m follow-up. Exclusion criteria were: (a) nurses in training, probationary, or short-term contractual positions; (b) individuals anticipated to be unavailable for follow-up due to maternity leave, extended sick leave, or other reasons. No sampling frame and no probability sampling were used: participants were recruited consecutively, as they entered employment, from 14 tertiary (grade-A) hospitals, and the number contributed by each site was small (average 21.1 participants per hospital). Because participants were therefore nested within hospitals, the multilevel structure of the data was examined explicitly before the models were specified.

For cross-lagged panel analysis combined with structural equation modeling, sample size was estimated following established guidelines. Kline recommends a minimum of 200 cases for structural equation models (Kline, 2018), with at least 10 observations per estimated parameter. The present study measured three core variables across three time points, with 23 total scale items. Applying the 10-fold rule yielded a minimum requirement of 230 cases; accounting for an anticipated 20% attrition rate in longitudinal tracking, the target sample size was set at 288.

### Measurement instruments

General information questionnaire. A self-designed questionnaire collected demographic data (gender, age, marital status, parental status) and employment-related information (employment type, monthly income, department, monthly night shifts).

Psychological Contract Breach Scale. Developed by (Robinson and Wolfe Morrison 2000) and revised into Chinese by Huang and Huang (2018), this unidimensional scale comprises 5 items rated on a 5-point Likert scale (1 = strongly disagree to 5 = strongly agree), with 3 items reverse-scored. Total scores range from 5 to 25, with higher scores indicating greater perceived breach. The Chinese version demonstrated a Cronbach's α of 0.89 and a content validity index of 0.93. In the present study, Cronbach's α values were 0.833, 0.796, and 0.850 at T1, T2, and T3, respectively.

Career Compromise Scale. Developed by Weng et al. (2018), this instrument contains 12 items across three dimensions: developmental opportunity compromise (4 items), career fit compromise (4 items), and social expectation compromise (4 items). Items are rated on a 5-point Likert scale, yielding a total score range of 12–60, where higher scores reflect greater compromise. The scale reported a Cronbach's α of 0.910. In this study, α values were 0.882, 0.920, and 0.824 at the three time points.

Turnover Intention Scale. Originally developed by Michaels and Spector (1982) and translated into Chinese by Lee and Lee (2003), this 6-item scale covers three dimensions: likelihood of quitting (2 items), intention to search for alternative employment (2 items), and perceived ease of finding external employment (2 items). Items use a 4-point Likert format (1 = never to 4 = frequently), with total scores ranging from 6 to 24. The Chinese version reported a Cronbach's α of 0.824. In this study, α values were 0.805, 0.814, and 0.852.

## Procedure and quality control

Data collection followed a strictly scheduled protocol. The T1 baseline survey was conducted between August and December 2024 within the first month of employment. Follow-up assessments (T2, T3) were administered at 6-month and 12-month intervals, corresponding to February–June 2025 and August–December 2025. All surveys were distributed electronically via the Wenjuanxing platform.

Anonymity was preserved throughout. At T1, participants created a unique identifier using the last six digits of their national ID number; this code was required to access T2 and T3 surveys, ensuring data linkage across waves. Six research team members received unified training on inclusion criteria and response protocols. All questions were mandatory; submissions from a single IP address were limited to one per survey; completion times under 120 s were flagged as invalid; and two attention-check items were embedded to detect careless responding. Following each wave, two researchers independently performed double-entry verification, and incomplete follow-up questionnaires were excluded.

At T1, 389 questionnaires were distributed, yielding 367 valid responses (effective rate: 94.34%). At T2, 321 valid responses were retrieved (attrition: 12.53%). At T3, 295 valid responses were obtained (cumulative attrition: 19.62%). A total of 295 cases were retained for cross-lagged analysis, satisfying the sample-size requirements for structural equation modeling.

## Statistical analysis

Descriptive statistics and preliminary analyses were performed using SPSS 26.0. Normally distributed continuous variables are reported as mean ± standard deviation; repeated-measures analysis of variance was used to examine changes over time. Categorical variables are summarized as frequencies and percentages. Pearson correlation analysis assessed bivariate associations.

Measurement invariance across time points was tested in Mplus 8.3 using the criteria ΔCFI ≤ 0.01 and ΔTLI ≤ 0.01. A three-wave cross-lagged panel model was then constructed to examine longitudinal associations and their temporal ordering. Model fit was evaluated using the chi-square-to-degrees-of-freedom ratio (χ^2^/df), Comparative Fit Index (CFI), Tucker–Lewis Index (TLI), Root Mean Square Error of Approximation (RMSEA), and Standardized Root Mean Square Residual (SRMR), with thresholds of χ^2^/df <3, CFI > 0.90, TLI > 0.90, RMSEA <0.08, and SRMR <0.08 indicating acceptable fit.

Model comparison followed an explicitly nested logic. The autoregressive baseline model was nested within each unidirectional model, which in turn was nested within the integrated model; the two unidirectional models, by contrast, were not nested with respect to each other. All models were estimated with maximum likelihood (ML) in Mplus 8.3; the same ML estimator was used for model estimation, for the nested comparisons and for the bootstrap resampling described below, so nested models were compared with the standard chi-square difference test (Δχ^2^ evaluated on Δdf = df(restricted model) – df(unrestricted model)) (Bentler and Dudgeon, 1996), with no scaling correction. Because M2 and M3 are not nested with respect to each other, they were compared on relative fit indices (CFI, TLI, RMSEA and SRMR). Both the hypothesized and the reverse cross-lagged paths were freely estimated in the integrated model, and significant and non-significant coefficients alike are reported so that readers can evaluate the directionality of the effects. Directionality was additionally tested formally: for each reciprocal pair of cross-lagged paths, the equality of the two standardized coefficients was evaluated with the Wald test implemented in the Mplus MODEL TEST command (Wald χ^2^ on 1 df), so that any claim that a hypothesized path exceeds its reverse counterpart is tested rather than assumed.

Because observations were clustered within 14 hospitals (average 21.1 participants per site), clustering was examined before the models were interpreted. Intraclass correlation coefficients were computed for the three T1 variables and were all below 0.05, giving design effects [1 + (m – 1)·ICC] below 2.0; the final model was accordingly estimated at the individual level and then re-estimated using the TYPE = COMPLEX option in Mplus, which adjusts standard errors and the chi-square statistic for non-independence (Muthén and Satorra, 1995). The two sets of estimates were substantively identical, so the individual-level results are reported.

Sensitivity of the cross-lagged estimates to the modeling of stability was considered explicitly. The traditional cross-lagged panel model does not separate stable between-person differences from within-person change, so its cross-lagged coefficients may partly reflect trait-like, between-person associations. A random-intercept cross-lagged panel model (RI-CLPM), which adds random intercepts to absorb time-invariant between-person variance, would address this directly (Mulder and Hamaker, 2021). Such an analysis was not conducted within the pre-specified analytic plan; we therefore retain the CLPM estimates as the primary results, interpret them strictly as temporal predictive associations, and record the omission as an explicit limitation rather than as evidence about within-person processes.

Attrition was examined in two ways. Participants who completed all three waves were compared with those who dropped out on baseline demographic characteristics and, additionally, on the T1 scores of the three study variables, using independent-samples t tests (or Mann–Whitney U tests where the normality assumption was violated); Little's MCAR test was used to evaluate the missing-data mechanism (Little, 1988). Because a complete-case analysis can be biased when data are missing at random (MAR) rather than completely at random, the final model was additionally re-estimated with full-information maximum likelihood (FIML) in Mplus, which retains all available observations under the MAR assumption [36]; the pattern of significant paths was unchanged. Neither Little's MCAR test nor FIML can exclude a missing-not-at-random (MNAR) mechanism, and because all three variables increased monotonically across the waves, dropout associated with high turnover intention is a plausible MNAR scenario; the implications of this possibility are set out in the Limitations. To reduce the threat of common method bias procedurally, the three waves were separated by 6 month intervals, and anonymity, reverse-scored items and attention checks were used; the corresponding statistical checks are reported in the Results section.

The longitudinal mediating effect of career compromise was tested using Mplus's built-in Bootstrap procedure (5,000 iterations) to compute 95% confidence intervals. Indirect effects whose 95% CIs excluded zero were considered statistically significant. Gender, age, employment type, and department were included as covariates. A two-tailed P <0.05 was considered statistically significant.

## Results

### Participant characteristics

A total of 295 Master of Nursing graduates completed all three waves. Comparison between dropouts (*n* = 72) and retained participants (*n* = 295) on general characteristics revealed no significant differences (*P* > 0.05). Because attrition may still be selective with respect to the study variables themselves, the two groups were also compared on their baseline (T1) scores; these comparisons were likewise non-significant (psychological contract breach: *t* = 0.56, *P* = 0.575; career compromise: *t* = 0.46, *P* = 0.648; turnover intention: *t* = 0.48, *P* = 0.630), and Little's MCAR test did not reject the missing-completely-at-random assumption (χ^2^ = 21.356, df = 18, *P* = 0.262). Taken together, these analyses suggest—although they cannot prove—that attrition did not introduce substantial selection bias. Detailed participant characteristics are presented in Table 1.

**Table 1:** General characteristics of participants (*n* = 295).

Item	Category	n (%)	Item	Category	*n* (%)
Gender	Male	28 (9.49)	Employment	Tenured	52 (17.63)
	Female	267 (90.51)		Contract	184 (62.37)
Age (yr)	<25	174 (58.98)		Personnel agency	59 (20.00)
	≥25	121 (41.02)	Department	Internal med	73 (24.75)
Marital	Unmarried	247 (83.73)		Surgery	88 (29.83)
	status	Married	48 (16.27)		ER/ICU	57 (19.32)
Children		Yes	6 (2.03)		Other	77 (26.10)
	No	289 (97.97)	Night shifts	<3/month	273 (92.54)
Monthly	<5,000 CNY	95 (32.20)	(per month)	3–6	18 (6.10)
	income	5,000–10,000	146 (49.49)		>6	4 (1.36)
>10,000		54 (18.31)			

### Common method bias test

Harman's single-factor test was conducted separately for each wave. The largest extracted factors explained 15.63%, 24.12%, and 28.97% of the variance at T1, T2, and T3, respectively—all below the 40% threshold (Robinson and Wolfe Morrison, 2000). Because Harman's single-factor test is only a preliminary and relatively insensitive diagnostic (Tang and Wen, 2020; Podsakoff et al., 2003), these results should be interpreted with caution: they suggest, rather than demonstrate, that common method bias was unlikely to be a serious concern. Procedural remedies were additionally built into the design, including temporal separation of the measurement waves by 6 m intervals, assurance of anonymity, and the inclusion of reverse-scored items. We therefore also estimated a model containing an unmeasured latent method factor: relative to the final model, CFI changed from 0.985 to 0.987 (ΔCFI = 0.002, i.e., below the 0.01 threshold) and the standardized factor loadings shifted by only 0.01–0.04, indicating that the substantive factor structure was essentially unchanged.

### Changes in study variables over time

Repeated-measures ANOVA revealed significant upward trends across all three variables during the 12-month follow-up: psychological contract breach (*F* = 68.373, *P* < 0.001), career compromise (*F* = 92.153, *P* < 0.001), and turnover intention (*F* = 54.822, *P* < 0.001). Detailed scores are shown in Table 2.

**Table 2:** Scores of psychological contract breach, career compromise, and turnover intention across three time points (*n* = 295, mean ± SD).

Variable	T1	T2	T3
Psychological Contract Breach	14.45 ± 2.26	15.21 ± 2.41	15.90 ± 2.35
Career Compromise	30.36 ± 5.74	33.54 ± 5.05	34.75 ± 5.97
Turnover Intention	15.24 ± 2.14	16.33 ± 2.53	16.91 ± 2.36
F-value	68.373	92.153	54.822
*P*-value	<0.001	<0.001	<0.001

### Correlation analysis

Pearson correlation analysis showed that all three variables exhibited significant positive autocorrelations across T1–T3 (*r* = 0.403–0.577, all *P* < 0.01). Within each time point, psychological contract breach was positively correlated with career compromise (*r* = 0.625–0.662), psychological contract breach with turnover intention (*r* = 0.580–0.616), and career compromise with turnover intention (r = 0.650–0.708), all at *P* < 0.01. These patterns satisfy the prerequisites for subsequent cross-lagged panel analysis. The full correlation matrix is presented in Table 3.

**Table 3:** Correlation matrix of psychological contract breach, career compromise, and turnover intention across three time points (*n* = 295).

Variable	PCB T1	CC T1	TI T1	PCB T2	CC T2	TI T2	PCB T3	CC T3	TI T3
PCB T1	1	0.625^**^	0.582^**^	0.486^**^	0.422^**^	0.385^**^	0.403^**^	0.366^**^	0.330^**^
CC T1		1	0.650^**^	0.411^**^	0.512^**^	0.458^**^	0.356^**^	0.440^**^	0.408^**^
TI T1			1	0.362^*^	0.449^**^	0.531^**^	0.320^*^	0.389^**^	0.469^**^
PCB T2				1	0.662^**^	0.616^**^	0.477^**^	0.459^**^	0.413^**^
CC T2					1	0.708^**^	0.434^**^	0.541^**^	0.499^**^
TI T2						1	0.391^**^	0.487^**^	0.577^**^
PCB T3							1	0.632^**^	0.580^**^
CC T3								1	0.676^**^
TI T3									1

PCB, psychological contract breach; CC, career compromise; TI, turnover intention. ^*^P < 0.01, ^**^P < 0.001.

### Measurement invariance testing

Configural, weak, and strong invariance models were sequentially tested in Mplus 8.3. For each variable, all constrained models showed ΔCFI ≤ 0.01 and ΔTLI ≤ 0.01 relative to the configural baseline, confirming that the measurement instruments possessed cross-time comparability. Detailed fit indices are reported in Table 4.

**Table 4:** Measurement invariance testing results across three time points.

Variable	Model	χ^2^	df	CFI	RMSEA (95% CI)	SRMR
PCB	M1	692.766	364	0.962	0.049 (0.043–0.056)	0.049
	M2	708.781	375	0.955	0.050 (0.042–0.054)	0.050
	M3	723.693	386	0.952	0.049 (0.045–0.057)	0.048
CC	M1	815.934	460	0.964	0.048 (0.044–0.054)	0.047
	M2	843.965	472	0.962	0.051 (0.046–0.055)	0.049
	M3	860.416	496	0.959	0.049 (0.043–0.054)	0.046
TI	M1	299.182	126	0.952	0.060 (0.052–0.066)	0.058
	M2	312.110	133	0.955	0.058 (0.051–0.062)	0.055
	M3	336.684	142	0.948	0.056 (0.052–0.066)	0.053

PCB, psychological contract breach; CC, career compromise; TI, turnover intention; M1 = configural model; M2 = weak invariance (equal factor loadings); M3 = strong invariance (equal intercepts).

### Cross-lagged panel model

Four candidate models were evaluated before selecting the final analytic model. M1 was a baseline model containing only autoregressive paths and within-wave correlations. M2 added the hypothesized cross-lagged paths (psychological contract breach → career compromise, psychological contract breach → turnover intention, career compromise → turnover intention), and M3 added the corresponding reverse paths (career compromise → psychological contract breach, turnover intention → psychological contract breach, turnover intention → career compromise). M4 was an integrated model that estimated both sets of cross-lagged paths simultaneously. M1 is nested within M2, M3, and M4, and M2 and M3 are nested within M4; M2, and M3 are not nested with respect to each other. Fit indices and model comparison results, including SRMR for every model, are summarized in Table 5.

**Table 5:** Model fit and comparison results for cross-lagged panel models.

Model	χ^2^	df	χ^2^/df	CFI	TLI	RMSEA	SRMR	*Δχ*^2^ (Δdf) vs. M1
M1	2133.563	435	4.90	0.941	0.910	0.093	0.089	—
M2	1235.127	364	3.39	0.972	0.965	0.040	0.051	898.436(71)^*a*^
M3	1296.253	342	3.79	0.966	0.943	0.041	0.054	837.310(93)^*a*^
M4	712.425	293	2.43	0.985	0.965	0.022	0.036	—

M1 = baseline model; M2 = hypothesized-direction model; M3 = reverse-direction model; M4 = integrated model. χ^2^/df = chi-square/degrees of freedom ratio; CFI, comparative fit index; TLI, Tucker–Lewis index; RMSEA, root mean square error of approximation; SRMR, standardized root mean square residual. M1 is nested within M2, M3 and M4, and M2 and M3 are nested within M4; M2 and M3 are not nested with respect to each other. ^*a*^Chi-square difference test versus M1, evaluated on Δdf = df(M1) – df(model); *P* < 0.001. For the nested comparisons involving M4: M4 vs. M2, Δχ^2^ = 522.702, Δdf = 71; M4 vs. M3, Δχ^2^ = 583.828, Δdf = 49 (both *P* < 0.001). All models were estimated with maximum likelihood (ML) in Mplus 8.3; Δχ^2^ is the standard ML chi-square difference statistic evaluated on Δdf, and no scaling correction was applied.

The baseline model (M1) showed inadequate fit against the thresholds stated above (χ^2^/df = 4.90, RMSEA = 0.093) and was rejected. Because M1 is nested within M2 and M3, the two were compared with M1 using the ML chi-square difference test described above (Δχ^2^ = χ^2^(M1) – χ^2^(model), evaluated on Δdf); both fitted significantly better than M1 (M2 vs. M1: Δχ^2^ = 898.436, Δdf = 71; M3 vs. M1: Δχ^2^ = 837.310, Δdf = 93; both *P* < 0.001). Neither model, however, fitted as well as the integrated model: both still exceeded the χ^2^/df <3 criterion adopted here (3.39 and 3.79, respectively) and showed higher RMSEA (0.040 and 0.041) and lower CFI (0.972 and 0.966) than M4, and M3 additionally showed a lower TLI (0.943) than M2 (0.965). Because M2 and M3 are not nested with respect to each other, they were compared on these relative fit indices rather than by a chi-square difference test, which favored M2. The integrated model (M4), within which both M2 and M3 are nested, showed a further improvement in fit over both (M4 vs. M2: Δχ^2^ = 522.702, Δdf = 71; M4 vs. M3: Δχ^2^ = 583.828, Δdf = 49; both P <0.001; ΔCFI = 0.013 and 0.019, respectively; χ^2^/df = 2.43, CFI = 0.985, TLI = 0.965, RMSEA = 0.022). M4 was therefore selected as the final model.

In the final cross-lagged model (Figure 1), all autoregressive paths from T1 to T3 were significant (coefficients 0.49–0.58, all *P* < 0.01; career compromise T2 → T3, β = 0.50), indicating moderate temporal stability. Psychological contract breach significantly predicted subsequent career compromise (T1 → T2: β = 0.25, *P* < 0.001; T2 → T3: β = 0.26, P <0.001) and subsequent turnover intention (T1 → T2: β = 0.18, *P* < 0.001; T2 → T3: β = 0.16, P <0.01). Career compromise likewise significantly predicted subsequent turnover intention (T1 → T2: β = 0.22, *P* < 0.001; T2 → T3: β = 0.21, *P* < 0.01). Because the integrated model estimated both the hypothesized and the reverse cross-lagged paths, all of the cross-lagged coefficients are reported in Table 6—including the reverse paths and those that did not reach statistical significance—so that the directionality of the effects can be evaluated. All coefficients are standardized and adjusted for gender, age, employment type and department.

**Figure 1:**
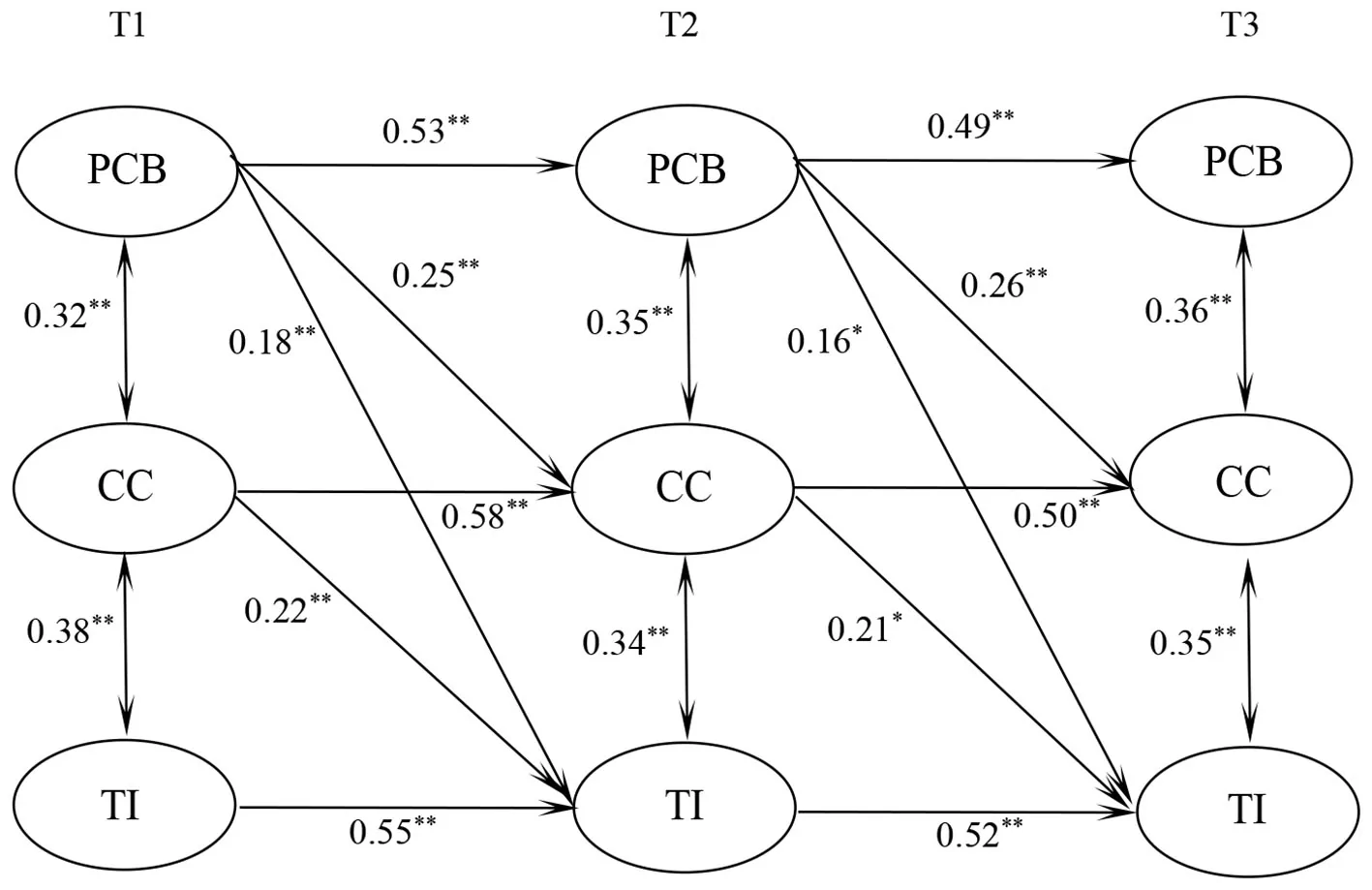
Cross-lagged panel model of psychological contract breach, career compromise, and turnover intention among newly recruited Master of Nursing graduates. *P < 0.01, **P < 0.001. Solid arrows indicate significant paths (*P* < 0.05). T1 = baseline (within 1 month of employment); T2 = 6 months; T3 = 12 months. PCB = psychological contract breach; CC = career compromise; TI = turnover intention. All autoregressive paths were significant at *P* < 0.01. Reverse cross-lagged paths estimated in the model are not displayed in the figure for clarity; all estimated cross-lagged paths are reported in Table 6.

**Table 6:** All cross-lagged paths estimated in the integrated model (M4).

Cross-lagged path	T1 → T2 β (P)	T2 → T3 β (P)
PCB → CC	0.25 (<0.001)	0.26 (<0.001)
PCB → TI	0.18 (<0.001)	0.16 (<0.01)
CC → TI	0.22 (<0.001)	0.21 (<0.01)
CC → PCB (reverse)	0.06 (0.312)	0.07 (0.268)
TI → PCB (reverse)	0.05 (0.394)	0.04 (0.451)
TI → CC (reverse)	0.08 (0.196)	0.06 (0.283)

PCB, psychological contract breach; CC, career compromise; TI, turnover intention. Values are standardized coefficients with *P* values in parentheses, adjusted for gender, age, employment type and department. Paths labeled “reverse” test the opposite direction of prediction. All estimated cross-lagged paths are reported regardless of statistical significance so that the directionality of the effects can be assessed. The reverse paths were small and statistically non-significant (*P* > 0.05), whereas the hypothesized paths were all significant—a pattern consistent with, though not by itself sufficient to establish, the hypothesized temporal ordering. Formal tests of the difference between each hypothesized coefficient and its reverse counterpart are reported in Table 7.

Formal tests of directionality. Because the integrated model estimates each cross-lagged path jointly with its reverse counterpart, the temporal ordering was tested rather than assumed: for every reciprocal pair, the equality of the two standardized coefficients was evaluated with the Wald test implemented in the Mplus MODEL TEST command (Wald χ^2^ on 1 df). The results are given in Table 7. All six differences favored the hypothesized direction, ranging from Δβ = 0.12 to Δβ = 0.19. The difference was statistically significant for breach → career compromise over the T1–T2 interval (Δβ = 0.19, Wald χ^2^(1) = 3.88, *P* = 0.049) and borderline over the T2–T3 interval (Δβ = 0.19, Wald χ^2^(1) = 3.53, *P* = 0.060); the remaining four comparisons did not reach significance (Δβ = 0.12–0.15, Wald χ^2^(1) = 2.16–2.63, *P* = 0.105–0.142). With standard errors of roughly 0.06 for each standardized coefficient, the power of these pairwise tests is limited, so a non-significant result should not be read as evidence that the two directions are equivalent. The conclusion is therefore narrower than one based on comparing significance status across paths: the reverse paths were uniformly small and statistically indistinguishable from zero, the hypothesized paths were all significant, and every point estimate favored the hypothesized ordering—but the pairwise differences are imprecisely estimated, and we do not claim that directionality has been established for every pair.

**Table 7:** Wald tests of the equality of each reciprocal pair of cross-lagged coefficients (integrated model, M4).

Reciprocal pair	Interval	β (hypothesized direction)	β (reverse direction)	Δβ	Wald χ^2^(1)	*P*
PCB → CC vs. CC → PCB	T1 → T2	0.25	0.06	0.19	3.88	0.049
PCB → TI vs. TI → PCB	T1 → T2	0.18	0.05	0.13	2.63	0.105
CC → TI vs. TI → CC	T1 → T2	0.22	0.08	0.14	2.36	0.124
PCB → CC vs. CC → PCB	T2 → T3	0.26	0.07	0.19	3.53	0.060
PCB → TI vs. TI → PCB	T2 → T3	0.16	0.04	0.12	2.16	0.142
CC → TI vs. TI → CC	T2 → T3	0.21	0.06	0.15	2.30	0.129

PCB, psychological contract breach; CC, career compromise; TI, turnover intention. Δβ = β (hypothesized direction) – β (reverse direction). Wald χ^2^ statistics have 1 degree of freedom and were obtained with the Mplus MODEL TEST command under maximum likelihood estimation; coefficients are standardized and adjusted for gender, age, employment type and department.

Bootstrap mediation analysis (5,000 iterations) revealed one significant indirect pathway from T1 psychological contract breach to T3 turnover intention. It runs sequentially through career compromise at T2 and is the product of the T1 → T2 cross-lagged path from breach to career compromise (β = 0.25) and the T2 → T3 cross-lagged path from career compromise to turnover intention (β = 0.21): 0.25 × 0.21 = 0.053, 95% CI = [0.032, 0.112], *P* < 0.01. No second pathway is reported: within-wave associations between the constructs are specified in this model as residual covariances rather than as directional paths, and a covariance has no direction and cannot form a leg of an indirect effect. Inference is therefore restricted to the single pre-specified pathway, and neither a total indirect effect nor a proportion-mediated statistic is reported. The 95% CI excluded zero, which is consistent with a longitudinal mediating role of career compromise; consistent with the observational nature of the design, this indirect effect should be interpreted as a temporal association rather than as a causal effect.

Statistical significance should not be equated with practical importance. Converting the standardized indirect effect back into the original metric (SD of T1 breach = 2.26; SD of T3 turnover intention = 2.36) gives approximately 0.053 × (2.36/2.26) = 0.055 turnover-intention points per point of breach. Taking the observed mean rise in breach from T1 to T3 (15.90 – 14.45 = 1.45 points) as the exposure contrast, the implied increase in turnover intention attributable to this pathway is about 0.08 points, which is less than 5% of the observed 1.67-point rise in turnover intention over the same period (15.24 to 16.91). The indirect effect is therefore small: it establishes the presence and the direction of an early-career process, not its magnitude.

## Discussion

### Upward trends in psychological contract breach, career compromise, and turnover intention

Xu et al. (2025) noted that early-career turnover among Master of Nursing graduates contributes to workforce discontinuity, stagnation of specialty development, and erosion of nursing innovation capacity. The present longitudinal data corroborate this concern: all three variables rose significantly over the first 12 months of employment. This pattern likely reflects a mismatch between the elevated career expectations of advanced-degree nurses and the realities of hospital human-resource systems (Ring and Hult, 2025).

Master of Nursing graduates typically enter the workforce with systematically cultivated research skills and specialty expertise, anticipating roles in advanced clinical practice, nursing management, or research. However, many tertiary hospitals continue to rely on traditional linear promotion and rotational systems. New graduates are often rapidly assimilated into standard clinical schedules, assigned substantial amounts of basic care and repetitive tasks, while research platforms and structured career pathways remain scarce. This environment fosters a sense of underutilization and developmental constraint, producing cumulative feelings of psychological contract breach (Gong and Sims, 2023).

As breach perceptions intensify, individuals adopt defensive strategies—lowering career standards and adjusting expectations—to maintain cognitive balance and psychological homeostasis. This manifests as elevated career compromise, which in turn is accompanied by weaker professional identity and organizational belonging and by higher turnover intention (Qiao et al., 2023). Interpreted cautiously, these findings indicate that the first year is a period in which the psychological contract is actively renegotiated and career expectations are revised downward. Because job characteristics, promotion systems and organizational resources were not measured in this study, we do not infer that any specific structural intervention is supported by our data. What the data do support is a narrower and testable proposition: breach and perceived compromise are inexpensive to monitor during the first months and may help identify nurses at risk, and interventions aimed at the fulfillment of developmental promises—rather than at pay or working conditions alone—are the ones most consistent with the mechanism observed here. Whether such interventions reduce compromise or turnover remains an empirical question requiring a controlled design.

### Interrelationships among the three core variables

The autoregressive coefficients (0.49–0.58) indicate substantial rank-order stability: relative standing on all three constructs was largely preserved across the 12 months. Two consequences follow. First, the cross-lagged coefficients are estimated on the residual, time-specific portion of each construct, which is why they are considerably smaller than the concurrent correlations reported in Table 3 (0.58–0.71); this attenuation is an expected property of the model and not evidence that the associations are weak ^[11]^. Second, such stability implies that a substantial part of the association among breach, compromise and turnover intention may be trait-like and between-person rather than a within-person process unfolding over time—a point taken up in the Limitations.

The strong correlation between psychological contract breach and career compromise suggests that when nurses perceive shortfalls in organizational resource allocation, job placement, or developmental commitments, they tend to down-regulate career expectations and accept suboptimal placements as a means of avoiding cognitive dissonance. This passive adaptation represents an individual-level behavioral response to organizational breach (Xie et al., 2024).

The strongest correlation was observed between career compromise and turnover intention, indicating that salary adjustments or environmental improvements alone are insufficient to offset the departure impulse triggered by constrained career development. When highly educated nurses are compelled to perform work misaligned with their capabilities over prolonged periods, their turnover intention intensifies most sharply (Zhou et al., 2023). These are concurrent correlations and cannot establish that constrained development causes turnover intention. They do, however, suggest that perceived developmental constraint is more closely tied to departure intentions than pay or working conditions in this sample, which makes it a reasonable candidate target for subsequent intervention studies rather than a demonstrated lever.

How do these estimates compare with prior evidence? Direct comparison is difficult, because few studies have modeled the same three constructs longitudinally and because cross-lagged estimates depend heavily on the measurement interval and on whether stability is controlled. Two benchmarks are nevertheless informative. First, in the meta-analytic literature on nurses, psychological contract and its breach show moderate concurrent associations with turnover intention (Lu et al., 2023); the cross-lagged estimates obtained here are markedly smaller (β = 0.16–0.18 for breach → turnover intention), which is consistent with the interpretation that much of the cross-sectional association reflects stable, between-person components that autoregressive control removes (Hamaker et al., 2015). Second, meta-analytic evidence indicates that behavioral intentions correlate only about *r* = 0.50 with actual turnover (Steel and Ovalle, 1984), so translating the present estimates into expected resignations would attenuate them further still. By these benchmarks the present coefficients are of a magnitude typical for three-wave panel studies of work attitudes and should not be read as large effects.

### Psychological contract breach as a temporal precursor with cross-time stability

The cross-lagged analysis indicated that psychological contract breach significantly predicted later career compromise and turnover intention, and that career compromise in turn predicted later turnover intention; given the observational design, these findings should be read as evidence of temporal precedence rather than of causality. The pattern of path coefficients from T1 to T3 suggests that organizational failure to honor commitments to highly educated nurses may trigger enduring cognitive restructuring. When newcomers discover that actual job content diverges sharply from pre-employment expectations regarding research, teaching, or specialty development, they avoid sustained psychological conflict by lowering standards and suppressing aspirations, resulting in career compromise (Cui and Huang, 2025).

By point estimate, the path from career compromise to turnover intention was stronger than that from psychological contract breach, although the two did not differ significantly (Table 7); career compromise occupies the position of a bridge in the temporal chain from organizational breach to turnover intention in this sample. When highly educated nurses are compelled to engage in prolonged mismatched mechanical work, their sense of professional accomplishment may be severely undermined, and a deeper sense of relative deprivation may emerge (Wang and Zhou, 2022), which may lead them to view turnover as a means of restoring professional dignity and self-efficacy.

Furthermore, the cross-lagged coefficients were consistently larger over the T1–T2 interval than over the T2–T3 interval. The “vulnerable window” reading — that the first six months are the period of greatest plasticity — is one explanation, but several alternatives must be weighed. (i) The two intervals are not equivalent in meaning even though both span 6 months: T1 was completed during onboarding, when expectations are still largely anticipatory, whereas T2 and T3 follow assignment to a stable post, so the T1–T2 contrast captures the transition from anticipated to experienced conditions. (ii) Regression to the mean cannot be excluded: T1 ratings were given before exposure to the workplace and may be less well calibrated, so part of the T1–T2 association may reflect measurement rather than change. (iii) Seasonal and institutional cycles differ between the windows: T2 fell between February and June and T3 between August and December, so the T3 assessment follows the year-end appraisal, contract-renewal and bonus cycles, which may add construct-irrelevant variance to the T3 measures. (iv) Selective attrition would compress variance at T3 and attenuate the later paths. (v) Adaptation may occur: nurses who remain beyond 6 months may have partly accommodated, so further compromise translates less strongly into intentions. These accounts are not separable with three waves; distinguishing them requires more closely spaced and more evenly distributed measurement occasions.

### Longitudinal mediating role of career compromise

The direct path from T1 psychological contract breach to T2 turnover intention was significant, indicating that early contract breach was directly associated with stronger subsequent departure impulses. The indirect pathway identified above further illuminates the role of career compromise.

The indirect pathway—through T2 career compromise—suggests that higher T1 breach was followed by greater T2 compromise, which in turn was associated with higher T3 turnover intention. This suggests that when highly educated nurses are forced to lower professional standards to accommodate organizational realities, such passive concessions may be transformed into active departure motivation (Pyhäjärvi and Söderberg, 2024).

Career compromise was also highly stable over time: its T2 → T3 autoregressive coefficient in the final model was 0.50, so nurses who had already revised their career expectations downwards at 6 months tended still to report high compromise at 12 months. This persistence matters because it makes an early downward revision unlikely to resolve itself without attention. It cannot, however, be converted into a second indirect effect here, because within-wave associations are modeled as residual covariances rather than directional paths. Whether persistent compromise adds to turnover intention over and above the cross-lagged pathway reported above requires a model in which that association is given a directional and theoretically justified specification—for example a random-intercept or latent-growth formulation—and remains a question for future research.

Within the limits set out above, the most these data support is a monitoring implication rather than a prescription. It may be worth attending to the early-career positioning of Master of Nursing graduates and to the alignment between assigned duties and professional expertise during the first months, and to evaluating in a controlled design whether competency assessment and job-matching mechanisms reduce career compromise. Whether adjusting job responsibilities prevents the entrenchment of career compromise, or reduces turnover, is not established by observational data of this kind and requires such an evaluation rather than assumed here.

## Conclusion

Psychological contract breach, career compromise, and turnover intention among Master of Nursing graduates all increase significantly during the first year of employment, and stable longitudinal associations exist among them. Psychological contract breach was not only directly associated with subsequent turnover intention but was also indirectly associated with it through career compromise at 6 months. Because the data are observational, these temporal associations do not establish causality, and the indirect effect is small. Within these bounds, and for newly recruited Master of Nursing graduates in comparable Chinese tertiary hospitals, the findings identify breach of developmental promises and emerging career compromise as plausible early-career signals; whether those signals are modifiable, and whether modifying them changes retention, is not established by these data and requires controlled evaluation.

This study has several limitations that should be considered when interpreting the findings.

First, the traditional cross-lagged panel model applied here does not separate stable between-person differences from within-person change. The cross-lagged coefficients may therefore partly reflect trait-like, between-person associations rather than within-person change over time, and should not be interpreted as causal effects. This concern is not merely formal here: the autoregressive coefficients of 0.49–0.58 show that the constructs are strongly trait-like, so a substantial share of the cross-lagged association is likely to be between-person. A random-intercept cross-lagged panel model (RI-CLPM), which explicitly separates between-person stability from within-person dynamics, would provide the more stringent test; that sensitivity analysis was not conducted, and we therefore acknowledge explicitly that the findings describe temporal predictive associations rather than causal or within-person effects. Future studies with comparable three-wave (or denser) panel data are encouraged to re-examine these associations using RI-CLPM or related within-person models (Mulder and Hamaker, 2021).

Second, external validity is limited in several respects. Participants were recruited by convenience sampling from 14 tertiary hospitals; no sampling frame and no probability design were used and the number contributed per site was small. The sample was also highly homogeneous: 90.5% were women, 83.7% were unmarried, 98.0% had no children and 59.0% were younger than 25 years. The findings therefore cannot be extrapolated to male nurses, to nurses with caring responsibilities, to nurses in secondary or primary hospitals, or to other regions of China, and the implications drawn above are scoped accordingly to newly recruited Master of Nursing graduates in comparable tertiary hospitals; they are stated throughout as temporal associations, not as effects that would follow from intervening on these variables. In addition, although the ICCs were small and the clustered re-analysis agreed with the individual-level results, between-hospital differences in human-resource practice were not modeled and may contribute unmeasured heterogeneity.

Third, attrition over the 12-month follow-up was not negligible (cumulative attrition: 19.62%). Although retained participants and dropouts did not differ significantly on baseline socio-demographic characteristics or on the baseline levels of the three study variables, and Little's MCAR test did not reject the missing-completely-at-random assumption (χ^2^ = 21.356, df = 18, *P* = 0.262), these checks cannot exclude selective dropout related to unmeasured characteristics. More important, this pattern of results is also compatible with a missing-not-at-random (MNAR) mechanism: because all three variables rose monotonically, nurses with the highest turnover intention may have been the most likely to leave the study, and neither Little's MCAR test nor the FIML re-analysis reported above can distinguish MAR from MNAR. The primary analyses were based on complete cases (n = 295), and although the FIML re-analysis reproduced the pattern of significant paths, this only addresses efficiency under MAR, not bias under MNAR. If dropout was indeed related to turnover intention, the reported increases and the T2 → T3 paths are likely to be conservative.

Fourth, all constructs were assessed by self-report at each wave. Harman's single-factor test is only a preliminary and relatively insensitive diagnostic of common method bias; even with the supplementary latent-method-factor check reported above, the conclusion that common method bias was unlikely to be serious should be regarded as provisional, and social desirability responding cannot be entirely excluded. Multi-source or multi-method designs, for example incorporating supervisor ratings, would help to address this concern.

Fifth, the outcome was turnover intention rather than actual turnover. Meta-analytic evidence indicates that behavioral intentions correlate only about r = 0.50 with actual turnover ^[35]^, so the intention–behavior gap is substantial: intention is a necessary but far from sufficient precursor of leaving, and the translation from intention to resignation is moderated by labor-market conditions, contract terms and personal circumstances that were not measured here. The practical significance of these findings therefore cannot be inferred from the coefficients alone. Future follow-up linking survey data to objective resignation or employment records would be required before any claim about retention outcomes.

Sixth, the observation window comprised 12 months and only three measurement occasions. Such a design can establish temporal ordering but cannot characterize the shape of change: non-linear trajectories, turning points and “tipping points” in the breach–compromise–intention sequence cannot be detected, and a 6 month spacing may be too coarse to locate the timing of the underlying processes. Describing trajectories rather than transitions will require designs with more closely spaced waves.

Seventh, this study examined only the mediating role of career compromise and did not consider potential moderating variables such as perceived organizational support, psychological capital, or leadership style. Future research should construct more complex multivariate interaction models to further elucidate the underlying mechanisms of turnover among highly educated nurses.

## Data Availability

The original contributions presented in the study are included in the article/supplementary material, further inquiries can be directed to the corresponding authors.
